# An Attempt at Matching Waking Events Into Dream Reports by Independent Judges

**DOI:** 10.3389/fpsyg.2018.00465

**Published:** 2018-04-06

**Authors:** Jia Xi Wang, He Yong Shen

**Affiliations:** Center for Studies of Psychological Application, School of Psychology, South China Normal University, Guangzhou, China

**Keywords:** continuity hypothesis, content analysis, dreams, incorporation, metaphor, memory consolidation, search activity

## Abstract

Correlations between memories and dreaming has typically been studied by linking conscious experiences and dream reports, which has illustrated that dreaming reflects waking life events, thoughts, and emotions. As some research suggests that sleep has a function of memory consolidation, and dreams reflect this, researching this relationship further may uncover more useful insights. However, most related research has been conducted using the self-report method which asks participants to judge the relationship between their own conscious experiences and dreams. This method may cause errors when the research purpose is to make comparisons between different groups, because individual differences cannot be balanced out when the results are compared among groups. Based on a knowledge of metaphors and symbols, we developed two operationalized definitions for independent judges to match conscious experiences and dreams, the descriptive incorporation and the metaphorical incorporation, and tested their reliability for the matching purpose. Two independent judges were asked to complete a linking task for 212 paired event-dreams. Results showed almost half dreams can be matched by independent judges, and the independent-judge method could provide similar proportions for the linking task, when compared with the self-report method.

## Introduction

Memory consolidation refers to the stabilization and integration of information into long-term memory networks (Marr, 1970). This may be measured either by an increase in performance in a memory task (enhancement) or a lack of a reduction in performance (maintenance).

Over the years, research has suggested that sleep has a function in memory consolidation; this has been shown in molecular, cellular, neurophysiological, brain-imaging studies (for a review, see Stickgold, 2005). Besides, dreams can also provide a window into the activity of the sleeping brain, as shown by other research.

Many dream studies have been carried out, trying to explore potential correlations between participants’ conscious experiences and dream reports. Research has consistently identified certain factors which increase the likelihood of a conscious experience appearing in dreams (review see Horton and Malinowski, 2015), such as an event’s emotional significance (e.g., Malinowski and Horton, 2014) and how recently it occurred (Blagrove et al., 2011; van Rijn et al., 2015). Building on these findings, Horton and Malinowski (2015) proposed an AM model to describe and explain the construction of dreams. In this model, autobiographical memory (AM) experiences are broken down into constituent fragments, reactivated ‘offline’ (during sleep), and recombined into a novel experience via ‘hyper-associativity.’ This refers to the increased activation of weakly semantically related concepts and networks, following the activation of a specific concept or memory (Stickgold et al., 1999; Llewellyn, 2013). Dreams reflect this process. AM, as mentioned above, is a “memory of the events of one’s life” including personal semantic information (e.g., knowing one’s own name) and personal episodic information (e.g., remembering a first date) (Baddeley, 1992). According to the AM model, dreaming extracts experiences or emotions from waking life and then cements them into perceivable scenes, during which the process of recombining relies on the connection of weakly semantically related memories. As dreams reflect this process, it seems there should be some semantic associations between conscious experiences and dream reports. In the area of semantics, metaphors can help to establish correspondence between concepts from disparate domains of knowledge (for a review, Bowdle and Gentner, 2005). Therefore, we can imagine that there are dream symbols that help to connect dream content with waking experiences. This idea is supported by researchers who note that dreams can be metaphors for waking life, picturing conscious experiences and emotions in non-literal, figurative ways (e.g., Jung, 1948a,b; Lakoff, 1993; Hartmann, 1996; review see Malinowski and Horton, 2015). Dream metaphors may reflect the semantic associations of the recombined process in the AM model. Coincidently, Hall and Nordby (1972) notes that there are two kinds of dreams, denotative dreams and metaphorical dreams. Denotative dreams directly represent their corresponding conscious experience, while metaphorical dreams represent something less obvious, and may express complex, even contradictory ideas. Denotative dreams do not require any kind of ‘decoding’ to understand their conscious life referent, whereas metaphorical dreams do.

As research suggests that insights and benefits can be obtained from the consideration of the relationship between a waking source and a dream content (Hill et al., 1998; Edwards et al., 2013, 2015), creating a method to decode dreams could be important and helpful. Up to now, dream research has mostly studied correlations between conscious experiences and dream reports using the self-report method (e.g., Malinowski and Horton, 2014), in which participants link events with dreams themselves, while only a few used an independent-judge method (e.g., van Rijn et al., 2015), which required independent judges to link participants’ events with dreams. When compared to the independent-judge method, the self-report method had an advantage in that the dreamer can recall and perceive more overlaps between conscious experiences and dreams. However, as different participants may have different abilities to judge potential correlations of events-dreams, this self-report method may cause potential errors which may not be balanced out when a study aims to explore whether some personality traits can affect conscious experiences’ incorporation into dreams. E.g., As mentioned above, emotional experiences were found to be preferentially incorporated into dreams (e.g., Malinowski and Horton, 2014), thus it can be anticipated that people with high neuroticism may have more waking events incorporated into dreams than people with low neuroticism, for neuroticism was a trait related to negative emotions (e.g., Karreman et al., 2013). Yet if the neuroticism is a factor that can affect the ability to judge potential correlations of events-dreams, the self-report method would produce errors which can not be counterbalanced between different trait groups, when study the correlation between trait neuroticism and the likelihood of a conscious experience appearing in dreams. By contrast, the independent-judge method asked external judges to match all participants’ conscious experiences to dreams, based on operational definitions that have already been formed, thus it can reduce errors caused by different trait groups, for all the matching would be done by a similar criteria (a same independent judge).

The main difficulty for the independent-judge method is creating operational definitions for external judges to use to identify any relationships between the conscious experiences and dream reports. As mentioned above, dream metaphors can help to connect conscious experiences and dream contents. So, the question is: what factors are at work in creating a dream metaphor. In other words, what guides a conscious event to become incorporated into a dream. In line with sleep and memory research (e.g., Payne and Kensinger, 2010; Wamsley and Stickgold, 2011), Malinowski and Horton (2014) propose that emotional memories are preferentially activated during sleep, thus appearing in dreams, in order to assimilate these memories into the wider memory system. This idea suggests that emotions can work as a trigger for incorporating events into dreams. However, several research suggest that external judges underestimate emotions of dreams when compared with dreamers (e.g., Schredl and Doll, 1998). So if external judges look for emotions when linking conscious experiences with dreams, the independent-judge method may not be able to bring the same number of linkages as the self-report method, for which the dreamer can feel the correlation rather than analyze it. This may cause a ‘floor effect’ which means independent judges could hardly recognize any correlations of events-dreams across all participants’ reports. Thus other elements for the linkage were needed for external judges to match events into dreams.

According to cognitive appraisal theory by Lazarus (1991; review see Watson and Spence, 2007), emotions are extracted from our evaluations (appraisals or estimates) of events that cause specific reactions in different people. Essentially, our appraisal of a situation causes an emotional, or affective, response that is going to be based on that appraisal. This theory implies that cognitive experience can also be viewed as serving a function of connecting waking events with dreams. It has been suggested that dreams are composed narratives (e.g., Montangero, 2012), and narratives are suggested to be the “basic manner in which the brain organizes experiences” (Pace-Schott, 2013, p. 2). As narratives always contain a behavior which may produce an outcome, the key element to link conscious experiences with dreams may be the outcome of an action in an experience. This is because these outcomes represent one’s cognitive appraisal of episodic experiences. The behavioral outcome is the result of a (significant) situation, usually bringing either an advantage (e.g., to fulfill one’s desire, to solve a problem, etc.) or a disadvantage (e.g., to cause a danger, to let someone down, etc. Consider an example in Lakoff (1993): “A woman I will call Karen dreamt that she was in the class of her favorite professor in college. He came over to her and said that she wasn’t working and would fail the class.” Lakoff explained this dream by making an association between Karen’s waking life and the dream. “Karen had recently married a professor who was a colleague of the professor in the dream. When she got married she quit a job she had hated and was not then working. She feared that her not working would lead to financial pressures that would cause the marriage to fail. The dream expresses Karen’s fear that her marriage will fail because she quit her job.” From this case, we can see that the same behavioral outcome between conscious life and dreaming help to match events in dreams.

We suggested two steps for matching dream reports and waking events. The first step, checking for the potential related elements between an event and a dream (such as similar characters, objects, or actions). The second step, checking for the behavioral outcome of the event and the dream. The first step can help to recognize parts of constituent fragments which are broken down from the AM experiences. The second step can help to recognize the key element which guides an event into a dream, to get a more reliable result. Based on these two steps, we created two types of operational definitions: the descriptive incorporation and the metaphorical incorporation (see Materials and Methods for details). In order to test their reliability, linking attempts were made by independent judges to enable a view.

## Materials and Methods

This study was approved by the local research ethics committee, and all subjects gave written informed consent before the start of the study.

### Participants

Participants took part in the research, and recorded their conscious experiences and dreams, with an average age 21.93, *SD* = 2.28, from 18 to 26. Male 7, Female 64. They were either undergraduates or postgraduates in Colleges of Guangzhou province, China.

#### Waking Event Collection

Participants were asked to record their three categories of waking events and night dream reports. Waking events were divided into three categories, taken from Fosse et al. (2003): Major daily activities (MDAs): Activities that took up most of the participants’ time during the day (e.g., going to work or university, meals, shopping). Personally significant events (PSEs): Important daily events that may or may not have taken up much time (e.g., emotional events). Major concerns (MCs): Concerns or thoughts that participants had on their mind during the day that may not have taken up much time, but were still considered important to them (e.g., money problems, exam stress).

#### Dream Collection

The method of recording a dream diary was the same as recommended by Selterman et al. (2012) method: Describe everything in your dreams, with as much detail as possible: What happened, in what time frame, with whom, etc. Describe the cognitions, emotions, and behaviors you experienced in your dreams, as well as the cognitions, emotions and behaviors of all other parties included in your dreams (if evident to you). If it was a lucid dream, state so. Continue on the reverse side of this sheet if needed.

#### Operational Definitions for Dream Decoding

In the description level, descriptive dreams are dreams that directly express waking life’s events. Metaphorical dreams are dreams which express waking life’s events in an indirect way. Based on these definitions, two ways in which waking events are incorporated into dreams were distinguished and named: descriptive incorporation and metaphorical incorporation. Descriptive incorporation is the incorporation of conscious experiences into dreams in a direct way, and is being easily identified because both conscious experiences and dream reports shared the same behavior. Metaphorical incorporation is the incorporation of conscious experiences into dreams in an indirect way, which is filled with symbolic expressions. It required checking for some indirect expressions of its conscious referent incorporated into dreams by symbolic language.

Their operationalized definitions are in **Table 1**.

**Table 1:** Operational definition for different kinds of incorporation.

Category	Operational definition
Descriptive incorporation	Dream subject element (e.g. character or object) is the same as the waking event’s description, and both the behavior^b^ and the behavioral outcome^c^ of the dream are in accord with the behavior^b^ and the behavior outcome^c^ of that event.
Metaphorical incorporation	(i) Dream subject element (e.g., character or object) is the same as the waking event’s description, and the dream’s behavior^b^ is not the same as the waking life behavior^b^ but their behavioral outcomes are the same as each other. (ii) Dream subject element (e.g., character or object) shares a similarity^a^ with the waking life event’s description, and the behavioral outcome^c^ of the dream is in accord with the behavior outcome^c^ of that event. If either i or ii can be found out, it would be viewed as metaphorical incorporation.

*^a^Similarity means that two elements can be categorized as of the same taxonomy (e.g., a character and an animal are viewed as the same taxonomy ‘creature,’ while a character and a stone are not viewed as such). ^b^Behavior is the main action for a narrative event (e.g., for event ‘design for a homework,’ the behavior would be ‘design’). ^c^Behavioral outcome is the developmental consequence of a situation, usually producing either an advantage (e.g., to fulfill one’s desire, to solve a problem, etc.) or a disadvantage (e.g., cause a danger, let someone down, etc.).*

### Procedure

Participants were asked to record their dreams and waking experiences in a spreadsheet at home. As a reward, they could get feedback of their dream reports and a monetary reward. Due to the difficulty of decoding dreams, one pilot study was included in the dream diary experiment. Participants were asked to record events-dreams for 10 days. Eight people participated this pilot study. In the official test participants were asked to record events-dreams for 3 days. There were 63 participants in this experiment. Dream dairies and waking experiences were paired by the same day (day events and that night’s dream), regardless of their respective numbers (e.g., if four events were recorded in the conscious time and two dreams were reported in a single night, they were counted as one paired event-dream). Finally, we got 212 paired event-dreams. Then they were randomly arranged and coded by two independent raters who were blind to the subject variables. One rater was the author of this research himself and the other one was his schoolmate who had no experience of matching events into reports before. They were taught an example of dream metaphors, ‘Karen’s dream’ (Lakoff, 1993), which are shown in **Appendix 1**. After all independent raters understood it, they were asked to score each paired event-dreams by the operational definitions in **Table 1**. Since the potential task load for each independent judge was large, the linking task for paired event-dream had a stepwise design. In the first step, raters looked for the descriptive incorporation. In this step, if a single paired event-dream was rated as descriptive incorporation, the linking task for this pair would stop. Otherwise raters went on to the second step. In the second step, raters looked for the metaphorical incorporation. In this step, if the pair can be rated as metaphorical incorporation, the linking task for this pair would stop. Otherwise this pair would be rated as non-incorporation. After all the 212 paired event-dreams had been done by this stepwise design, judges examined whether those pairs having been rated as descriptive incorporation can be rated as metaphorical incorporation as well. Besides, considering MDAs seemed to have small incorporation in other research (e.g., Malinowski and Horton, 2014; van Rijn et al., 2015), in the present study only PSEs and MCs were scored by external judges. Finally judges counted the number of events (PSEs/MCs), and the length of dreams in each paired event-dream.

### Data Analysis

Two independent raters scored each types of incorporation for the total 212 paired event-dreams. The judges were asked to indicate with a ‘yes’ or ‘no’ if the dream contained mention of category 1 = descriptive incorporation; category 2 = metaphorical incorporation; category 3 = non-incorporation. A score of 0 for no presence or 1 for presence was used for analysis. Inter-rater reliability for judges’ initial rating scores was assessed. The Cronbach’s consistency coefficient were: α = 0.74. All inconsistent rating was discussed later carefully until reaching an agreement. Then the judges scored numbers of each types of incorporation having been reached an agreement. Due to the reason that a paired event-dream may come from more than one waking event, the judges also counted the number of PSEs’ incorporation and the number of MCs’ incorporation.

All statistical analysis methods above were performed in IBM SPSS 18.0 software.

## Results

### Descriptive Incorporation

In the present study, descriptive incorporation contained two ways for memories to be incorporated into dreams.

First, a replay of PSE with the same people or objects. e.g., PSEs: The dreamer was busy in dealing with questionnaire data. Dream: The dreamer was dealing with the questionnaire data.

Second, dreams reflected ones’ concerns by fulfilling related desires, with behavior that can bring about same behavioral outcome. e.g., MCs: The dreamer wanted to find a boyfriend. Dream: The dreamer was courted by someone attractive. In both the dream and the concern the character was the dreamer, and they had the same action ‘pursue,’ and shared the same behavioral outcome ‘a boyfriend’ to the dreamer.

### Metaphorical Incorporation

In the present study, metaphorical incorporation contained four ways in which memories could be incorporated into dreams.

First, dreams and conscious experiences’ behaviors were not the same but their behavioral outcome was the same, done by the same subject. e.g., PSE: The dreamer asked others for advice about work. Dream: The dreamer asked an old man for directions.

Second, dreams and conscious experiences’ behaviors were not the same but their behavioral outcome was the same, done by different characters. e.g., PSE: The dreamer did not finish a job of students’ communities. Dream: Someone failed to pass a test in the classroom. Both behaviors brought about the same outcome ‘failure’ to the dreamer.

Third, dreams reflected one’s concern by showing relative possibilities which can bring about the same behavioral outcome, done by the same subject. e.g., MC: The dreamer wants to lose weight. Dream: The dreamer went to a beauty salon to get a facial. Both the dream and the concern shared the same situation ‘beautifying’ to the dreamer.

Fourth, dreams reflected one’s concern by showing relative possibilities which can bring about the same behavioral outcome, done by other subjects. e.g., MC: The dreamer wants to know who I am. Dream: The dreamer met an animal who told the dreamer its name. In this case, the animal’s words indeed answered the dreamer’s concern with a simple answer.

### Descriptive Data of Events, Dreams, and Incorporation

The analyzed data set consisted of a total of 212 paired event-dreams, 155 from 61 participants in the 3 days experiment (mean 2.5 pairs per person), and 57 from 8 participants in the 10 days experiment (mean 7.1 pairs per person). The length of dreams and the number of PSEs/MCs are in **Table 2**.

**Table 2:** Average and median number of dream length and conscious event.

Content	Mean(*SD*)	Median
Dream length^a^	192 (136.2)	150
PSEs number^b^	1.49 (0.75)	1
MCs number^c^	1.52 (0.69)	1

*^a^Only words counted, including word ‘ums’, ‘uhs’ etc. ^b^The total number of PSEs is 316. ^c^The total number of MCs is 322.*

From the total of 212 paired event-dreams, except one was scored as both descriptive incorporation and metaphorical incorporation, almost all pairs was scored as either descriptive incorporation or metaphorical incorporation, of which 25 pairs were scored as descriptive incorporation, and 91 pairs were scored as metaphorical incorporation. Besides, 48 MCs and 77 PSEs were found to incorporate into dreams.

Frequencies data is shown in **Table 3**.

**Table 3:** The number of observations and frequencies for event’s incorporation.

Dream variable	Observations (percentage)
Non-incorporation^a^	97 (45.3%)
Descriptive incorporation^ab^	25 (11.8%)
Metaphorical incorporation^ab^	91 (43.0%)
PSEs’ incorporating^c^	77 (24.4%)
MCs’ incorporating^d^	48 (15.0%)

*^a^The total number of available paired event-dreams is 212. ^b^One paired event-dream was scored as both descriptive incorporation and metaphorical incorporation. ^c^The total number of PSEs is 316. ^d^The total number of MCs is 322.*

## Discussion

More than half (54.7%) dream reports were found to relate to conscious experiences. This is in accordance with Fosse et al. (2003) where 57% of the dream reports were fairly certainly caused by the conscious event. Besides, a proportion of 11.8% was found for the descriptive incorporation which meant that dreams directly reflect a waking event. This is in accordance with Dement et al. (1965) finding the replay of memories in dreams was 12% when scored by external judges. In the present study, the descriptive incorporation required judges to look for same characters or objects, and same behaviors and behavioral outcomes, between conscious experiences and dreams. In other words, if a paired event-dream was a descriptive incorporation, there would be at least two same features between the event and the dream. Correspondingly, Fosse et al. (2003) found that 11% of dreams and events contained the same location and at least two other features which can be emotions, themes, actions, characters or objects. In that research, a systematic search was performed for episodic memories in the dream reports, with the array of specific requirements based on the definition of episodic memory introduced in a stepwise manner. Participants kept a log of daytime experiences and dreams, and scored the dreams for incorporation of any conscious experiences. The only difference between the two pieces of research was that this study required external judges to match conscious experiences with dreams, whereas in that study the same task was done by participants themselves. These similar results suggest that using our operational definitions, independent judges are able to match participants’ conscious experiences with dreams.

Stickgold and Walker (2013) propose that for memories to be consolidated and integrated with existing knowledge, there is a process of memory triage that determines which memories should go through sleep-dependent processing and by which form of processing. Memory consolidation is thus a selective process, involving discriminatory processing of specific memories. Several studies suggest dreams reflect emotional experiences from conscious life (e.g., Schredl, 2006; Malinowski and Horton, 2014), and many studies also indicate that dreams generally reflect waking-life concerns (e.g., Domhoff, 2003). In the present study, both PSEs and MCs were found to be incorporated into dreams, which was in line with those previous studies. Furthermore, it was found that 24.4% PSEs were incorporated into dreams and a proportion of 15% for MCs’ incorporation. These proportions are higher than Malinowski and Horton (2014) where the proportion of PSEs’ incorporation was 12% and the proportion of MCs’ incorporation was 11%. This may be because in this study the specific operationalized definition for metaphorical incorporation was given, thus independent judges were more able to recognize metaphorical expressions, while in Malinowski and Horton (2014) the rating process was done by participants themselves who may miss some dream metaphors. Thus, the present study found more incorporations than that study. Future research can let participants score their own paired event-dreams by our operational definitions to study this issue.

In the present study, our operational definitions were created through the hypothesis that dream metaphors can help to establish correlations between waking events and dream reports, which can be seen as a semantically related relationship. After having searched for dream metaphors, results showed correlations between conscious experiences and dream reports. According to the dream continuity hypothesis, dreams express conscious concerns and emotional preoccupations (e.g., Domhoff, 2003, 2011; for a critical review, see Domhoff, 2017). More specifically, the comparison of dream content with waking life suggests that dreams express one’s conceptions of the people and activities that concern him in waking life, not merely one’s experiences in waking life. In this study the matching process were done by looking for the same behavioral outcome between waking experiences and dreams. This added to the dream continuity hypothesis, by showing that the advantageous consequence or the disadvantageous consequence for the dreamer can also help to establish correlations between waking experiences and dream content.

Rotenberg (2009) proposed that REM sleep is regarded as a specific form of search activity aimed at compensating for the lack of search in consciousness, and ensured the resumption of search activity in subsequent consciousness. The search activity is that activity in the uncertain situation with the constant feedback between behavior and its outcome. It manifests itself in the planning, fantasizing, and rethinking of the situation. The search activity can raise the body’s resistance to stress, to natural and experimentally induced pathology whereas renunciation of search may lead a non-specific predisposition to somatic disturbances (e.g. psychosomatic diseases). In the present study our operationalized definitions asked external judges to look for the behavioral outcome, this element was partly in accord with Rotenberg’s viewpoint that the search activity may bring metaphoric contents in dreams. Results showed that almost half-conscious experiences prior to sleep can be matched into dreams. Besides, among 212 paired event-dreams, only one pair was found to be both descriptive incorporation and metaphorical incorporation. By contrast, in some paired event-dreams, there were more than one waking event judged to incorporate into dreams in the metaphorical way. This may be because in the present study the descriptive incorporation may represent a disfunction of the search activity, and thus in this situation dream metaphors for other waking events would be hardly formed. These results may provide evidences for Rotenberg’s viewpoint.

In the present study there still remained nearly half of dream reports that were unable to recognize their conscious experiences. This may be because in the linking task paired event-dream was coming from the same day, which may lose potential results relative to time. In other words, this study did not explore the possibility that conscious experiences were incorporated into dreams at a later date. Previous research has shown the dream-residue effect which refers to the appearance in dreams of memory details from 1 or 2 days before, and the day-lag effect referring to a delayed incorporation of events into dreams 5–7 days after the event took place (e.g., Blagrove et al., 2011; van Rijn et al., 2015). Future research concerning matching for more days are welcomed, especially for addressing whether the independent-judge method can work out the dream-lag phenomenon, according to these operationalized definitions.

The present study aimed to create operational definitions available for external judges to do quantitative studies about dreams. Though the inter-rater consistency reliability of this research was less than the dream content analysis method based on the Hall and Van de Castle coding system (Hall and Van de Castle, 1966), they were chosen because the authors wished to quantify potential correlations between participants’ conscious experiences and dreams rather than only study dreams themselves. Results showed that for the purpose of linking conscious memories into dream reports, independent judges can provide similar effects to the linkage, compared to results from the self-report method. Thus, the qualitative matching attempt of this research proved the availability of our operational definitions. More research are needed to test their reliability for external judges to decode dreams.

### Limitation and Suggestion

In the present study, dreams were coded by independent raters depending on the operational definitions: descriptive incorporation and metaphorical incorporation. Thus a methodological issue must be considered. Up to now, the method for most research when exploring the correlation between waking events and dream reports may lead to a bias from raters’ self-attribution. That is the correlation may reflect raters’ belief in or ability to perceive overlaps between conscious memories and dreams, rather than overlaps *per se*. Nevertheless, this self-attribution may be balanced out when results would have come comparing between different groups (constant method). Therefore, in order to study the relationship between conscious experiences with dreams, a balanced design may help to reduce errors caused by the self-attribution.

In the present study, since the task load for external judges was large, only PSEs and MCs were scored to find their correlations with dreams, future research can study the correlation between participants’ MDAs and dreams. Besides, the linking task for external judges was a stepwise design. This may lose some results of PSEs/MCs’ incorporation. Because if a pair was found to be the metaphorical incorporation, the linking for this pair would stop, regardless correlations between other events and dream content in this pair. However, this design would hardly influence results of correlations between conscious experiences and night dreams, because each category of incorporations (non-/ descriptive-/metaphorical-) were only scored once for a paired event-dream, regardless the number of PSEs/MCs’ incorporation in the pair.

In the future it may require more judges for future work when using the independent-judge method, in order to guarantee the reliability. Because it was difficult to decode metaphorical dreams, though the decoding direction was given (operational definitions). Moreover, if possible, research should use both the self-report method and the independent-judge method to do the matching work, for the dreamer by himself may feel (without analyzing) some weak but important relationships between conscious experiences and dreams through their right hemisphere.

## Conclusion

The work reported here follows the suggestion by Malinowski and Horton (2015) that “Dream science can be particularly insightful in terms of the study of metaphors and associations between memory elements to reflect assimilation. It has been over 10 years since Domhoff’s (2003) suggestion that inductive qualitative methods, followed up with objective quantitative methods, are used to study dream metaphors in depth, but few researchers have broached this topic yet. More research is needed to investigate the conscious experiences of assimilation that occur during sleep in dreams, and dream metaphor, hyperassociativity, and bizarreness may provide starting points.” Based on the AM model and semantic metaphorical knowledge, this study created operational definitions for matching events in dreams and completed this matching process using independent judges. Descriptive results showed similar proportions for the matching attempt, when compared with the self-report method. Future research can use these operational definitions to make more quantitative research on dreams, especially on metaphorical dreams, and this may reveal more insight and benefits for the field.

## Author Contributions

JW contributed to the design of this research and finished it. HS contributed to submitting this paper to the journal and provided financial supports and language help.

## Conflict of Interest Statement

The authors declare that the research was conducted in the absence of any commercial or financial relationships that could be construed as a potential conflict of interest.

## Appendix 1

Dream: A woman I will call Karen dreamt that she was in the class of her favorite professor in college. He came over to her and said that she wasn’t working and would fail the class.

Event: Karen had recently married a professor who was a colleague of the professor in the dream. When she got married she quit a job she had hated and was not then working. She feared that her not working would lead to financial pressures that would cause the marriage to fail.

Association: The dream expresses Karen’s fear that her marriage will fail because she quit her job. First, Shared a similar element ‘professor,’ which worked as a mark for further checking, then the same behavioral outcome fail or fear was recognized and the analogy mechanism can be seen: ‘professor’ – ‘professor,’ ‘marry’ – ‘teach,’ ‘fail’ – ‘fail’ or ‘fear’ – ‘fear.’ Finally the wake-dream continuity of this dream was found out.
